# Concurrent beneficial (vitamin D production) and hazardous (cutaneous DNA damage) impact of repeated low‐level summer sunlight exposures†

**DOI:** 10.1111/bjd.14863

**Published:** 2016-11-18

**Authors:** S.J. Felton, M.S. Cooke, R. Kift, J.L. Berry, A.R. Webb, P.M.W. Lam, F.R. de Gruijl, A. Vail, L.E. Rhodes

**Affiliations:** ^1^ Dermatology Research Centre, Institute of Inflammation and RepairFaculty of Medical and Human SciencesUniversity of Manchester, Manchester Academic Health Science Centre, Salford Royal NHS Foundation TrustManchesterU.K.; ^2^ Oxidative Stress Group, Department of Environmental and Occupational HealthFlorida International UniversityMiamiFLU.S.A.; ^3^ School of Earth Atmospheric and Environmental SciencesUniversity of ManchesterManchesterU.K.; ^4^ Department of Clinical Biochemistry, Manchester Royal InfirmaryCentral Manchester NHS Foundation Trust, Manchester Academic Health Science CentreOxford RoadManchesterU.K.; ^5^ Oxidative Stress Group, Department of Cancer Studies and Molecular MedicineUniversity of LeicesterLeicesterU.K.; ^6^ Department of DermatologyLeiden University Medical CentreLeidenthe Netherlands; ^7^ Centre for Biostatistics, Institute of Population HealthUniversity of Manchester, Manchester Academic Health Science Centre, Salford Royal NHS Foundation TrustManchesterU.K.

## Abstract

**Background:**

The concurrent impact of repeated low‐level summer sunlight exposures on vitamin D production and cutaneous DNA damage, potentially leading to mutagenesis and skin cancer, is unknown.

**Objectives:**

This is an experimental study (i) to determine the dual impact of repeated low‐level sunlight exposures on vitamin D status and DNA damage/repair (via both skin and urinary biomarkers) in light‐skinned adults; and (ii) to compare outcomes following the same exposures in brown‐skinned adults.

**Methods:**

Ten white (phototype II) and six South Asian volunteers (phototype V), aged 23–59 years, received 6 weeks’ simulated summer sunlight exposures (95% ultraviolet A/5% ultraviolet B, 1·3 standard erythemal doses three times weekly) wearing summer clothing exposing ~35% body surface area. Assessments made were circulating 25‐hydroxyvitamin D [25(OH)D], immunohistochemistry for cyclobutane pyrimidine dimer (CPD)‐positive nuclei and urinary biomarkers of direct and oxidative (8‐oxo‐deoxyguanosine) DNA damage.

**Results:**

Serum 25(OH)D rose from mean 36·5 ± 13·0 to 54·3 ± 10·5 nmol L^−1^ (14·6 ± 5·2 to 21·7 ± 4·2 ng mL^−1^) in phototype II vs. 17·2 ± 6·3 to 25·5 ± 9·5 nmol L^−1^ (6·9 ± 2·5 to 10·2 ± 3·8 ng mL^−1^) in phototype V (*P* < 0·05). Phototype II skin showed CPD‐positive nuclei immediately postcourse, mean 44% (range 27–84) cleared after 24 h, contrasting with minimal DNA damage and full clearance in phototype V (*P* < 0·001). The findings did not differ from those following single ultraviolet radiation (UVR) exposure. Urinary CPDs remained below the detection threshold in both groups; 8‐oxo‐deoxyguanosine was higher in phototype II than V (*P* = 0·002), but was unaffected by UVR.

**Conclusions:**

Low‐dose summer sunlight exposures confer vitamin D sufficiency in light‐skinned people concurrently with low‐level, nonaccumulating DNA damage. The same exposures produce minimal DNA damage but less vitamin D in brown‐skinned people. This informs tailoring of sun‐exposure policies.

## Abstract 2

**What's already known about this topic?**

- Repeated low‐level exposures to simulated U.K. sunlight can produce vitamin D sufficiency in light‐skinned people, but the concurrent impact on cutaneous DNA damage is unknown.

**What does this study add?**

- Low‐level simulated sunlight exposures in people of skin phototype II conferred vitamin D sufficiency concurrently with DNA damage, which showed partial clearance at 24 h and no evidence of accumulated damage after 6 weeks of exposures.
- The same exposures produced minimal DNA damage but less vitamin D in brown‐skinned people (phototype V).
- The findings are informative for sun‐exposure guidance.

Plain language summary available online

Solar ultraviolet radiation (UVR) exposure has the established benefit to health of vitamin D synthesis, while skin cancer is a major hazard. Studies using various protocols have examined the impact of single‐ and repeated‐dose UVR on vitamin D status,^1^, ^2^, ^3^, ^4^, ^5^ but research examining accompanying UVR‐induced DNA damage is scarce. Recently, the impact of high‐intensity UVR exposures attained through a sunbathing holiday (Canary Islands, 28°N) on circulating 25‐hydroxyvitamin D [25(OH)D] and cyclobutane pyrimidine dimer (CPD) excretion in urine, as a proxy for UVR‐induced cutaneous DNA damage, was explored in white individuals.^5^ There were increases in both vitamin D status and urinary CPD, and the conclusion was made that under high‐level UVR exposure conditions, the vitamin D benefit is inevitably derived at the cost of DNA damage. However, this might differ with UVR exposure pattern and dose, and between phototypes.^6^, ^7^

Skin cancer is prevalent and causes a substantial health burden in white populations. The main exogenous risk factor, UVR, is a carcinogen, initiating DNA damage and suppressing skin immunity.^8^ UVB induces pyrimidine (6–4) pyrimidone photoproducts^9^ and CPDs,^10^ the dominant mutagenic form of direct UVR‐induced DNA damage,^11^ with thymine‐containing dimers being most common.^10^ If not repaired, these photoproducts form the ‘UVB signature’ mutations present in skin cancers.^12^ Recently, UVA was also shown to induce thymine‐containing dimers in human epidermis *in vivo*.^10^, ^13^ UVR also induces oxidatively generated damage to nucleic acids.^14^ UVR‐induced DNA damage stimulates melanogenesis, although this provides only modest protection against further UVR damage.^15^, ^16^ Urinary excretion of UVR‐induced DNA damage products may act as a convenient proxy for cutaneous DNA damage;^17^ however, to date, skin and urinary damage have not been directly compared.

UVB triggers conversion of 7‐dehydrocholesterol to previtamin D, the body's principal vitamin D source, with usually only small amounts obtained from diet.^18^ Vitamin D undergoes hepatic hydroxylation to 25(OH)D, the major circulating form and the current best indicator of vitamin D status, and subsequent renal hydroxylation to active 1,25‐dihydroxyvitamin D. There is associative evidence of diverse health benefits of vitamin D,^19^, ^20^, ^21^ while its established benefit is musculoskeletal, including prevention of rickets and osteomalacia.^22^, ^23^ Public health guidance recommends sun protection in individuals at high risk of skin cancer,^24^ while also considering vitamin D benefit. It is generally assumed that regular brief sun exposures to skin produce adequate vitamin D.^25^ Guidance is geared for light‐skinned individuals, and is supported by an intervention study in 109 white patients where simulated low‐level sunlight exposures, while they were casually dressed, produced vitamin D sufficiency, defined as 25(OH)D ≥ 50 nmol L^−1^ (20 ng mL^−1^).^1^

The objectives of this study were to examine the impact on cutaneous DNA damage/repair (skin and urinary biomarker assessment) alongside 25(OH)D gain with regular low‐level UVR exposures, in both white‐ and brown‐skinned people. We exposed 10 white and six South Asian volunteers to a simulated summer's brief exposures (95% UVA/5% UVB, three times weekly for 6 weeks). Skin biopsies were examined for CPD‐positive nuclei, for induction by a single 1·3 standard erythemal dose (SED) exposure, accumulation over 6 weeks’ UVR exposures, and clearance 24 h postcourse. Urine was analysed for CPD and 8‐oxo‐deoxyguanosine (8‐oxo‐dG) DNA damage.^26^ Through performance under known exposure conditions, the data gained are informative for sun‐exposure guidance.

## Patients and methods

### Patients

This was an experimental study in healthy volunteers. People of phototype II (white skin, sunburns easily, tans minimally) and phototype V (South Asian, brown skin), aged 18–60 years, from Greater Manchester, U.K. were recruited by advertisement (January 2010). Exclusion criteria were a history of skin cancer/photosensitivity, use of sunbed/sunbathing within 3 months, taking photoactive medication/vitamin D supplements, and pregnancy or breastfeeding. The North Manchester Research Ethics Committee provided ethical approval (reference 09/H1014/73). The study adhered to the Declaration of Helsinki; the volunteers gave written, informed consent.

### Minimal erythemal dose assessment

Individuals’ minimal erythemal doses (MEDs) were assessed precourse, as the lowest UVR dose producing a visually discernible erythema 24 h post‐UVR. A geometric series of 10 doses (7–80 mJ cm^−2^ for phototype II; 26·6–271 mJ cm^−2^ for phototype V) of erythemally weighted UVR was applied to buttock skin using a Waldmann UV236B unit with CF‐L 36W/UV6 lamps (peak emission 313 nm, range 290–400 nm; Waldmann GmbH, Villingen Schwenningen, Germany).

### Simulated summer sunlight exposures

Volunteers were given a 6‐week course of UVR exposures, concordant with the length of the U.K. school summer holiday, when the population is most exposed to sunlight, as described previously.^1^ A Philips HB588 Sunstudio irradiation cabinet (Philips, Eindhoven, the Netherlands) delivered whole‐body UVR exposure after fitting with alternating Arimed B (Cosmedico GmbH, Stuttgart, Germany) and Cleo Natural (Philips) fluorescent tubes, providing UVR emission close to U.K. summer sunlight (95% UVA: 320–400 nm, 5% UVB: 290–320 nm). Cabinet emission was characterized using a DTM300 spectroradiometer (Bentham, Reading, U.K.) and monitored using an Ocean Optics S2000 spectroradiometer (Ocean Optics, Dunedin, FL, U.S.A.). Wearing protective eye goggles, standardized T‐shirts and knee‐length shorts, volunteers lay prone, exposing ~35% skin surface in total.

UVR exposures of 1·3 SED were given three times weekly in January and February, when ambient UVB is negligible at U.K. latitudes and people are at trough vitamin D status,^27^ with exposure time adjusted to maintain constant dosing.^28^ Doses took ~6·5 min to administer, equating to 13–17‐min exposure to U.K. June midday sunlight exposure six times weekly, which takes account that (i) when horizontal, ventral and dorsal surfaces are exposed sequentially in sunlight, not simultaneously as in the cabinet; and (ii) in daily life, postures range from horizontal to vertical randomly orientated to the sun.^29^ To compare UVR‐exposed/protected sites, a 10 × 10‐cm^2^ aperture was made in the shorts material over one buttock; the contralateral buttock was covered with UVR‐opaque material.

### Dietary vitamin D logs

Volunteers completed daily dietary logs of vitamin D‐fortified foods, and six key food categories – cheese; butter, margarine and oily spreads; milk and milk‐containing products; red meat; oily fish; and eggs and egg dishes – during the first and last study weeks.^30^ Vitamin D content was obtained from food package labelling and *McCance and Widdowson's The Composition of Foods*.^31^

### Vitamin D, parathyroid hormone and serum biochemistry

Blood samples were taken weekly, and serum stored at −20 °C until study completion. Serum 25(OH)D was measured by high‐performance liquid chromatography–UV, as reported previously.^32^ The laboratory was accredited to ISO 9001:2008 and 13485:2003 standards, and certified proficient by the national vitamin D quality assurance scheme (DEQAS). Parathyroid hormone was measured at the beginning and end of the course, and serum biochemistry was analysed.^1^ Deficiency and sufficiency cut‐offs for 25‐(OH)D levels were defined as 25 nmol L^−1^ (10 ng mL^−1^) and 50 nmol L^−1^ (20 ng mL^−1^), respectively.^23^, ^33^, ^34^

### Skin colour measurements

UVR‐exposed and protected buttock skin colour was measured at baseline and weekly (CM‐2500d spectrophotometer; Konica Minolta, Tokyo, Japan). Triplicate standard L*a*b* data were recorded.^35^ The individual typology angle (ITA) was calculated as the vector direction in the L*b* plane, as arctangent [(L*−50)/b*] × (180/π).^36^, ^37^, ^38^

### Cutaneous sampling

Following the UVR course, all participants had four 4‐mm punch biopsies taken from buttock skin under the following conditions: photoprotected skin, immediately after 1 × 1·3 SED, immediately after 18 × 1·3 SED, and 24 h following the 18 exposures. Biopsies were formalin fixed and paraffin embedded for histological analysis.

### Cutaneous cyclobutane pyrimidine dimer immunostaining

Immunostaining was performed using a modification of the method of Tewari *et al*.^13^ 4‐μm sections were treated with 0·1% trypsin; hydrogen peroxide (0·3% in methanol) was added to inhibit endogenous peroxidase; and blocking buffer (Vector Laboratories, Peterborough, U.K.) was added, followed by monoclonal antibody incubation (TDM‐2, 1 : 2000; CosmoBio, Tokyo, Japan).^39^ The primary antibody was omitted from one slide/staining cycle as a negative control. Slides were incubated with biotinylated secondary antibody before addition of ABC solution, developed with Vector SG solution and counterstained with Nuclear Fast Red (Vector Laboratories) before dehydration and mounting. Images were scanned (Panoramic 250 Flash II; 3DHISTECH Ltd, Budapest, Hungary) and analysed for epidermal thickness and area (Image J 1.48; National Institutes of Health, Bethesda, MD, U.S.A.). Positively staining nuclei were counted per high‐power field (HPF) (original magnification ×40; 3 HPFs per section, 9 HPFs per slide). The researcher (S.J.F.) was blinded to the slide identity.

### Urinary analyses for DNA damage

First‐void urine samples were collected daily (Monday to Friday) during week 1 to assess for early impact, and then weekly to assess for accumulation of DNA damage. These were stored at −20 °C until processing.

### Quantification of urinary 8‐oxo‐deoxyguanosine

Samples were analysed for 8‐oxo‐dG using ultrahigh‐performance liquid chromatography (UHPLC)–tandem mass spectroscopy as described previously.^40^ The results were normalized using urinary creatinine.

### Quantification of urinary thymine dimers

We developed a UHPLC‐MS/MS assay for *cis*,* syn* T<>pT in urine, which benefits from stable isotope‐labelled internal standardization, is more rapid than the HPLC^32^P‐postlabelling method, avoids the need for 32P, and provides absolute quantification, unlike enzyme‐linked immunosorbent assay. CPDs are removed from DNA by nucleotide excision repair, as a lesion‐containing single‐stranded oligomer approximately 24–32 nucleotides long.^41^ These oligomers are subject to 5ʹ→3ʹ exonucleolytic attack, generating lesion‐containing 6‐ and 7‐mers, with some 2‐mers. The current methodology for measuring CPDs in urine is HPLC prepurification followed by 32P postlabelling. This approach quantifies the dimer as a dinucleotide monophosphate (the dimerized form of thymidylyl‐3ʹ‐5ʹ‐thymidine, T<>pT).^42^ However, potential exists for dimers to be present in urine as other oligomeric forms. Therefore we adopted two approaches: the first quantifies T<>pT, and the second utilizes formic acid hydrolysis of urine to render all oligomeric forms down to the nucleobase form of the dimer (thymine–thymine dimer, T<>T). These methods are detailed in Appendix S1 (see Supporting Information).

### Statistical analyses

Paired and unpaired *t*‐tests, repeated‐measures analyses and linear regressions were performed using SPSS statistical software (version 21.0.0; IBM, Armonk, NY, U.S.A.) and GraphPad Prism (version 6; GraphPad Software Inc., La Jolla, CA, U.S.A.). Ratio measures, logarithmically transformed to make them normally distributed, were considered statistically significant at *P* < 0·05.

## Results

### Volunteers

The volunteers were compliant with the study procedures and all completed the study. Table 1 displays their baseline characteristics; general serum biochemistry was normal. Baseline serum parathyroid hormone appeared lower (nonsignificantly) for phototype II than phototype V, and did not change significantly. Dietary vitamin D was low, with 80% of phototype II and 83% of phototype V volunteers ingesting < 5 μg per day, and was constant between weeks.

**Table 1:** Patient demographics

	Phototype II	Phototype V
Participants, *n*	10	6
Sex: male, female, *n*	2, 8	4, 2
Age (years)	45 ± 9	38 ± 11
Body mass index (kg m^−2^)	26 ± 4	26 ± 3
MED (mJ cm^−2^)	37 ± 13	146 ± 64
Baseline PTH (pmol L^−1^)^a^	2·3 ± 0·9	3·8 ± 1·7
Final PTH (pmol L^−1^)^a^	2·3 ± 0·7	3·1 ± 1·2
Dietary vitamin D intake week 1 (μg per day)	3·1 ± 2·7	2·6 ± 2·5
Dietary vitamin D intake week 6 (μg per day)	3·3 ± 2·6	2·0 ± 1·4
Baseline 25(OH)D (nmol L^−1^)	36·5 ± 13·0	17·2 ± 6·3
Final 25(OH)D (nmol L^−1^)	54·3 ± 10·5	25·5 ± 9·5

Values are the mean ± SD unless stated otherwise. MED, minimal erythemal dose; 25(OH)D, 25‐hydroxyvitamin D. ^a^The normal parathyroid hormone (PTH) range is 0·8–3·9 pmol L^−1^.

### Serum 25‐hydroxyvitamin D gain

**Figure 1:**
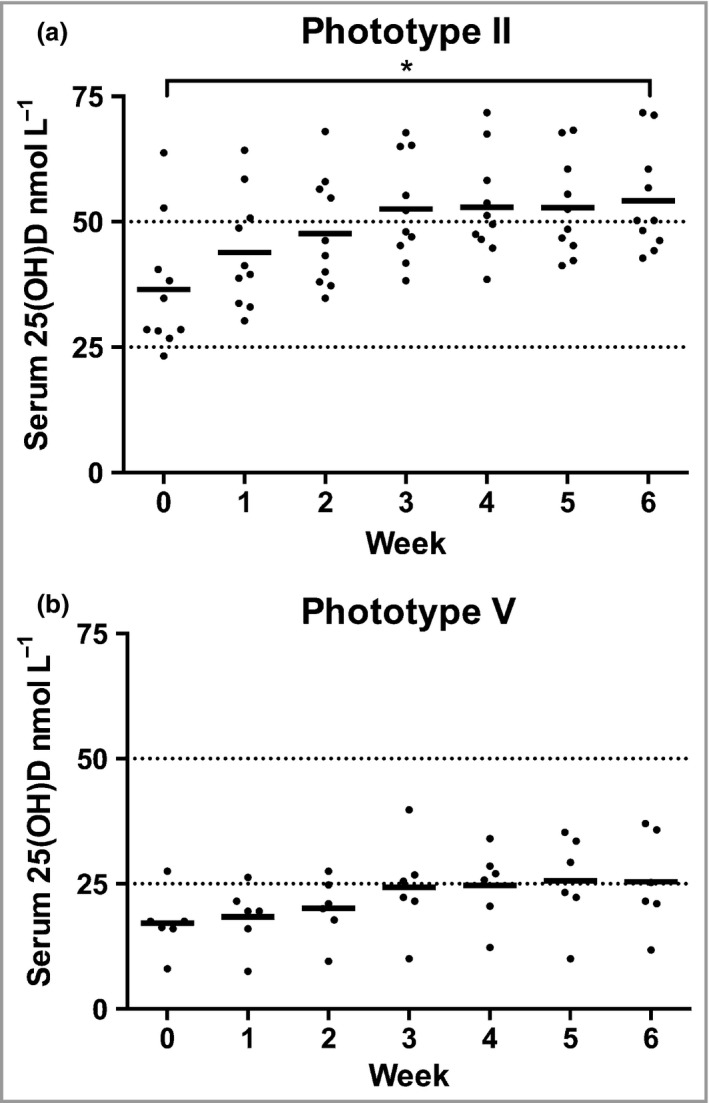
Levels of 25‐hydroxyvitamin D [25(OH)D] during the simulated summer sunlight exposures. Serum 25(OH)D increased during the 6‐week simulated summer ultraviolet radiation exposures, with a plateau in both groups around week 4. The values were significantly higher at all time points in individuals with phototype II (a; *n* = 10) than those with phototype V (b; *n* = 6). The 25(OH)D gain between baseline and week 6 was statistically significant for phototype II. Horizontal bars denote mean values, and horizontal lines represent the 25(OH)D level deficiency and insufficiency cut‐offs at 25 and 50 nmol L^−1^, respectively. *P < 0·001.

The 6‐week course produced a greater mean serum 25(OH)D gain in phototype II volunteers: 17·8 ± 4·8 nmol L^−1^ vs. 8·3 ± 10·5 nmol L^−1^ for phototype V (*P* < 0·05; Fig. 1). The gain was inversely associated with baseline 25(OH)D for phototype II (*R* ^2^ = 0·4; *P* = 0·049) but not phototype V. However, the proportional gain in 25(OH)D from baseline was almost identical, with a mean increase of 49% in phototype II, from 36·5 ± 13·0 at baseline to 54·3 ± 10·5 nmol L^−1^ at course end, and 48% from 17·2 ± 6·3 to 25·5 ± 9·5 nmol L^−1^ in phototype V. The post‐UVR level was positively associated with baseline 25(OH)D (*P* < 0·001), consistently with previous studies.^1^, ^43^

### Skin darkening

At baseline, the mean L* (skin lightness) was 69 ± 2·8 in phototype II volunteers and 41 ± 12·8 in phototype V, with mean ITAs of 52 ± 5·7° and −22 ± 33·3°, respectively. The 6‐week exposures produced significantly greater darkening in volunteers with phototype V than in those with phototype II, as indicated by the reduction in L* (*P* = 0·02), although this did not reach significance for ITA. ITA decrease (darkening) was positively associated with 25(OH)D gain for phototype II (*R* ^2^ = 0·54, *P* = 0·016) but not phototype V volunteers, in whom there was wide interindividual variation in ITA and less 25(OH)D gain.

### Cutaneous cyclobutane pyrimidine dimers

**Figure 2:**
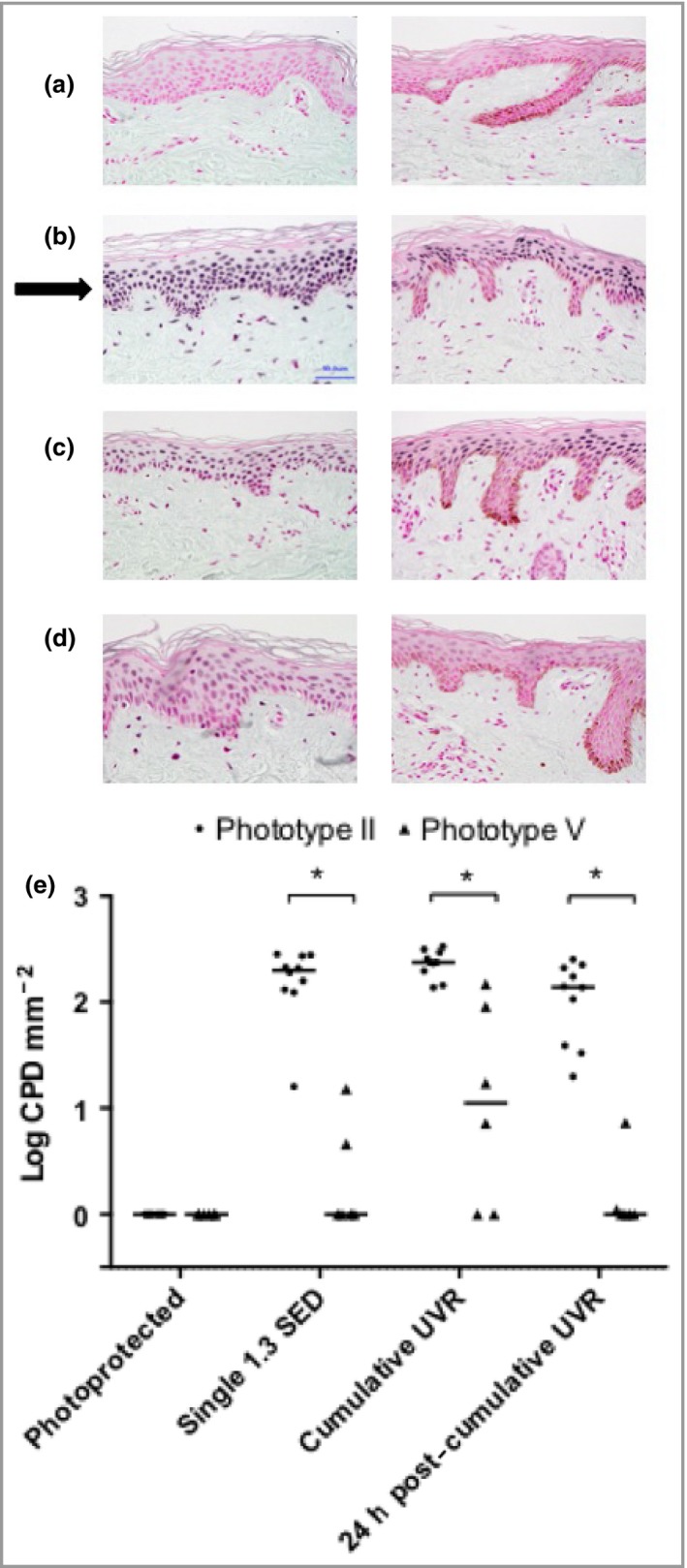
Representative epidermal DNA damage in individuals with phototype II and V skin under varying conditions of ultraviolet radiation (UVR) exposure. Cyclobutane pyrimidine dimer (CPD)‐positive nucleus staining (black arrow) from a volunteer of phototype II (left column) and phototype V (right column). Original magnification ×40. (a) Photoprotected skin; (b) immediately following one 1·3 standard erythemal dose (SED) exposure; (c) immediately following the completion of the 6‐week simulated summer sunlight exposures; (d) 24 h after the completion of the 6‐week simulated summer exposures. (e) CPD‐positive nucleus counts in volunteers with skin phototype II (circles; *n* = 10) and V (triangles; *n* = 6). DNA damage was absent from photoprotected skin in both groups. The median CPD‐positive nucleus counts were significantly higher in phototype II than V immediately following a single UVR exposure, after the 6‐week course of cumulative UVR exposures, and 24 h following the cumulative exposures (*P* < 0·001 for all). In both phototypes, the 6‐week simulated summer sunlight exposures caused no statistically significant difference in CPD‐positive nuclei compared with a single 1·3‐SED exposure. Horizontal bars denote the median. Viable epidermal thickness measurements did not differ between the two phototype groups, and were unchanged by the simulated summer sunlight exposures. *P < 0·001.

Skin‐section examination showed that UVR did not induce epidermal thickening in either phototype (data not shown). In the absence of UVR exposure, no CPDs were detectable in any individual (Fig. 2a, e). One 1·3‐SED exposure caused a range of CPD levels in phototype II individuals (median count 200 CPD‐positive nuclei mm^−2^, range 16·5–284; Fig. 2b), while only two phototype V volunteers showed any evidence of CPDs (counts of 4 and 16 CPD‐positive nuclei mm^−2^; Fig. 2e). Skin receiving cumulative UVR (18 × 1·3 SED) showed elevated CPD‐positive nucleus counts in phototype II (median 234 nuclei mm^−2^, range 125–314; Fig. 2c) vs. phototype V (median 12 nuclei mm^−2^, range 0–148, *P* < 0·001). No significant difference was seen in CPDs after cumulative vs. single exposure for either phototype. At 24 h after 6‐week exposures, phototype II volunteers had cleared a mean 44% (range 27–84%) of their cutaneous CPD‐positive nuclei, while those with phototype V had cleared 97% (range 84–100%, *P* < 0·001; Fig. 2d, e). Volunteers with phototype II showed a positive association of induction of CPD‐positive nuclei with baseline ITA (*R* ^2^ = 0·49; *P* = 0·02), but weak, nonsignificant associations with baseline L* (*R* ^2^ = 0·29), age (*R* ^2^ = 0·33) and 25(OH)D gain (*R* ^2^ = 0·23).

### Urinary DNA damage

**Figure 3:**
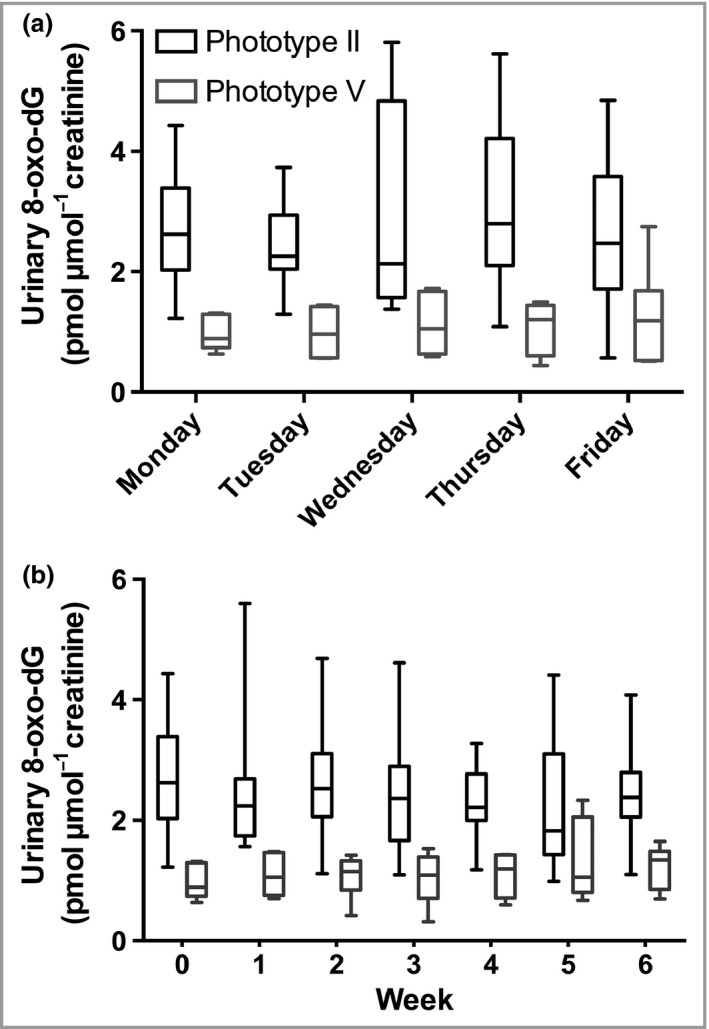
Urinary 8‐oxo‐deoxyguanosine (8‐oxo‐dG) damage (pmol μmol^−1^ creatinine) in volunteers with skin types II and V. Urinary 8‐oxo‐dG concentrations (a) daily for the first 5 days of week 1 and (b) weekly during the 6‐week simulated summer. Patients of phototype V (*n* = 6) had significantly lower 8‐oxo‐dG levels than those of phototype II (*n* = 10) both in the first 5 days (including prior to exposure) and during the study (*P* = 0·001 and *P* = 0·002, respectively, repeated measures). However, no increase in urinary oxidative DNA damage was seen at any of the time points following a single UVR exposure during the first 5 days, and no accumulation occurred over the 6‐week course. The data shown are the median, interquartile range and full range.

CPDs (T<>T and T<>pT) were undetectable for both phototypes, at baseline and after the UVR course. At baseline, phototype II volunteers had higher urinary 8‐oxo‐dG (mean 2·72 ± 0·97 pmol μmol^−1^ creatinine) than phototype V (mean 0·96 ± 0·28 pmol μmol^−1^ creatinine), *P* < 0·001, with no significant increase during any of the days measured in week 1 (Fig. 3a). Moreover, while 8‐oxo‐dG levels were higher in phototype II volunteers at all time points (repeated‐measures analysis, *P* = 0·001; Fig. 3b), there was no accumulation in urinary 8‐oxo‐dG over the 6‐week course.

## Discussion

To our knowledge, this is the first study examining the benefits and cutaneous DNA damage/repair effects of vitamin D concurrently following low‐level UVR exposures. Employing radiation similar to summer solar UVR emission and protocols simulating repeated casual exposures, UVR doses were equivalent to 13–17 min of U.K. June midday exposure, on most days of the week (latitude 53·5°N).^29^ Such exposures have been assumed to provide adequate vitamin D status, and were shown to provide serum 25(OH)D levels equating to sufficiency (50 nmol L^−1^)^23^ in white individuals.^1^ Assessment of concurrent DNA damage outcome (cutaneous and urinary) has awaited exploration.

We have now demonstrated that low‐level exposures readily induced CPDs in keratinocytes in white skin (phototype II) and, to a much lesser extent, in South Asian skin (brown, phototype V) *in vivo*. Induction was significantly positively associated with skin pallor (baseline ITA), consistent with a recent *ex vivo* human skin study.^38^ Comparison of DNA damage induced by one exposure to 1·3 SED with that following 18 doses revealed close similarity. There was no evidence for regular low‐level exposures leading to DNA damage accumulation, indicating effective repair between exposures.

Sheehan *et al*.^44^ described accumulation of CPD‐positive nuclei with repeated 0·65‐MED exposures in skin phototypes II and IV. However, this involved an MED‐adjusted dose, not and absolute UVR dose, and exposures at shorter intervals (Monday to Friday for 2 weeks). Another human study reported that it took 48–72 h for CPD‐positive nucleus levels to return to baseline following a single higher (1·2‐MED) exposure.^45^ In human keratinocytes *in vitro*, following low‐level UVB exposure (8 mJ cm^−2^, around twofold lower than the phototype I MED) on eight consecutive days, very few CPD‐positive nuclei had been repaired 24 h post‐UVR.^46^ Similarly, mice given repeated low‐level UVB (0·5 kJ m^−2^ every 24 h for 40 consecutive days) showed that CPD repair lagged behind formation, leading to damage accumulation.^9^ The low‐level UVR we employed may cause insufficient DNA damage to overwhelm repair,^44^, ^47^ or the 48‐h intervals between exposures could provide sufficient repair time. It is also feasible that repair mechanisms are upregulated by repeated low‐level exposures.

As CPD persistence can lead to mutagenesis, and repair kinetics in human skin are most rapid within 24 h,^48^ we quantified CPD‐positive nuclei in biopsies taken 24 h post‐UVR. In phototype II skin, a mean 44% of CPD‐positive nuclei were cleared vs. virtually all (97%) in phototype V, where the initial level of damage was much lower. The cumulative UVR study of Sheehan *et al*.^44^ also found more complete repair in skin type IV than II at 1 week post‐UVR. The decrease in CPD‐positive nuclei we observed at 24 h showed significant interindividual variation within phototype II skin (27–84%). From a human health perspective, it was encouraging that CPDs did not accumulate over the UVR course; nevertheless, a substantial proportion of damaged cells were still present 24 h post‐UVR, and the potential remains for mutagenesis after each DNA‐damaging event.

Interestingly, following both single and repeated (18 sessions) low‐level UVR exposures, urinary CPDs remained below the detection limit, and oxidatively damaged DNA did not increase from baseline, in either phototype. Concurrent skin‐section analysis confirmed CPD induction, but lack of urinary CPD detection suggests that the damage was relatively small, and/or the number of cells affected was insufficient to generate a signal in urine. This conclusion is supported by the urinary 8‐oxo‐dG findings. Our previous study showed that urinary 8‐oxo‐dG increases 4 days following single, whole‐body suberythemal (15 J cm^−2^) UVA exposure *in vivo*,^17^ suggesting that our levels of UVR exposure (reflecting the UVR dose and surface area exposed) were insufficient to induce urinary 8‐oxo‐dG, a sensitive biomarker of oxidative stress. Intriguingly, phototype II skin had greater urinary 8‐oxo‐dG than phototype V at all time points, implying a non‐UVR explanation, such as differences in metabolism, repair and/or antioxidant intake; this warrants future exploration.

Studies examining the impact of melanin on vitamin D synthesis *in vivo* show conflicting results,^2^, ^4^, ^6^, ^7^, ^49^ potentially through differences in skin site, baseline 25(OH)D, UVR dose and UVR spectrum.^50^ We found that most phototype II participants reached vitamin D sufficiency [25(OH)D ≥ 50 nmol L^−1^], which is consistent with our larger sample of white volunteers.^1^ Over half of the South Asian volunteers achieved sufficiency, attaining ≥ 25 nmol L^−1^, but none reached ≥ 50 nmol L^−1^, as in a previous investigation in 15 South Asian patients.^30^ In addition to the higher constitutive pigmentation in phototype V skin, they had significantly greater skin darkening during the UVR course than those with phototype II, and this may be responsible for the lower 25(OH)D gain/plateau in phototype V. Facultative pigmentation includes involvement of higher epidermal levels,^51^ limiting UVB penetration to 7‐dehydrocholesterol and hence initiation of vitamin D synthesis.

A positive association between urinary T<>pT and 25(OH)D gain was reported following intense UVR exposures (mean 60–101 kJ m^−2^) during sun/ski holidays in individuals with skin phototypes I–IV.^5^ Liljendahl *et al*.^52^ also identified significantly increased urinary T<>pT 3–5 days after 2 days’ beach sunbathing in Sweden, with urinary DNA damage strongly correlating with personal UVR dosage (up to 1400 J m^−2^). These high‐dose exposures contrast with our brief suberythemal exposures, where the association between cutaneous CPD‐positive nuclei and 25(OH)D gain was weak and nonsignificant. Building on the present study, application of a dose range of low‐level UVR exposures could assess whether there are doses where vitamin D benefit is gained with minimal DNA damage in light‐skinned adults. Similarly, a dose range of higher‐level exposures^4^ could examine whether brown‐skinned individuals can achieve higher serum 25(OH)D gain, still with limited DNA damage.

The main strength of this study is the original, concurrent examination of cutaneous CPDs with urinary DNA damage biomarkers and 25(OH)D gain, following low‐level UVR exposure. This simulation of northerly‐latitude summer sunlight exposures employed UVR emission close to that of midday sunlight, and examined 25(OH)D gain after repeated exposures to commonly exposed skin sites. Completion of dietary logs indicated no alteration in vitamin D intake over the study. Future studies may explore the findings in a wider range of phototypes, using differing patterns of UVR and natural sunlight exposure.

Our findings indicate tailoring of public health policies on safe sun exposure for different phototypes. Brown‐skinned individuals who experience almost negligible DNA damage but generate low amounts of 25(OH)D could be advised on less limited sun‐exposure practice,^4^ while caution is required for phototype II individuals, as unrepaired cutaneous DNA damage was seen at 24 h following even the low UVR doses employed, in these easily burning individuals.

## Supporting information


**Appendix S1.** Supplementary materials and methods.

**Video S1.** Author video.
