# Supplementary material for: Concurrent beneficial (vitamin D production) and hazardous (cutaneous DNA damage) impact of repeated low‐level summer sunlight exposures
Source: Br J Dermatol. 2016 Nov 18;175(6):1320–8. doi: 10.1111/bjd.14863 (PMC5215649; doi:10.1111/bjd.14863)
Supplement: Supplementary file 1 — Appendix S1. Supplementary materials and methods. [file BJD-175-1320-s001.docx]

**Supplementary material**

**Quantification of urinary thymine dimers by UHPLC-MS/MS**

Materials

Acetonitrile (HPLC gradient grade), water (HPLC gradient grade) and mass spectrometry grade formic acid were purchased from Fisher Scientific (Loughborough, UK). Ammonium formate (>99.0%) and acetophenone were purchased from Sigma-Aldrich (Poole, UK). Isotopically labelled ([^15^N_2_, ^13^C_10_]; CK Gas Products, Ibstock, UK) and unlabelled thymidylyl-(3’-5’)-thymidine (dTpdT) were synthesised by Carbosynth Limited (Berkshire, UK). Oasis WAX 50mg (Waters Corporation, Manchester, UK), and ISOLUTE ENV+ 50mg (Biotage, Uppsala, Sweden) solid-phase extraction (SPE) cartridges were purchased from their manufacturers.

Preparation of unlabelled and stable isotope labelled thymine dimers

Due to possible loss of analyte during sample workup, isotopically labelled internal standards were necessary for each target compound. The synthesis of both labelled (α,α,α,6-^2^H_4_) and unlabelled T<>T was based on the method of Wang (1961), as described by Podmore *et al* (1996). A frozen aqueous solution of thymine (2mg/mL) was stored at -80 °C for 12h prior to being UVC irradiated (254 nm UVS-18 EI series UV lamp) on dry ice for 4h. The solution was then thawed and the resulting precipitate isolated by centrifugation (1200 x g) for 20min, and then dried at 40°C. This method has been shown previously to produce a standard free from impurity by thymine (Podmore *et al.,* 1996).

Labelled thymidylyl-3’-5’thymidine (TpT) was custom synthesized by Carbosynth Ltd (Compton, UK) from [^13^C_10_, ^15^N_2_]-dThy. Only one molecule of dThy per TpT was labelled. Labelled ([^13^C_10_, ^15^N_2_]-T<>pT) and unlabeled T<>pT were generated by UVR exposure in the presence of a photosensitizer, based upon the method of Liu and Yang (1978). Oxygen was purged from a solution of TpT (5mg/22.7mL) in HPLC gradient grade water, and 13.2mM acetophenone, by bubbling nitrogen through the solution. The solution was then UVB irradiated (Philips TL-01 lamp) for 3.5h under nitrogen, at 10°C. The resulting photoproducts (*cis-syn* and *trans-syn* T<>pT) were isolated in the Department of Chemistry (University of Leicester) using preparative, reversed phase HPLC consisting of a Dionex Ultimate 3000 system and a Phenomenex Gemini NX 5µm C18 110Å AXIA column (250 x 21.20mm). Separation was achieved during a 35min isocratic run with 99.9% water and 0.1% trifluoroacetic acid, with a flow rate of 10.6mL/min. Only two peaks were seen in the chromatogram at 13.9 and 18.3min, both peaks were collected and subsequently freeze dried, the resulting yield was 2.7mg and <1mg respectively.

Urine pre-treatment and solid phase extraction

All urine samples were kept frozen at -20 °C until analysis, when they were thawed at room temperature and then centrifuged. Any pellet was discarded and the supernatant used in the subsequent steps. For T<>T, urine supernatants (10µL) were spiked with internal standard prior to formic acid hydrolysis, and solid phase extraction on ISOLUTE ENV+ SPE columns. Following SPE, the resulting eluate was evaporated to dryness under nitrogen before reconstitution in 50µL mobile phase. For T<>pT, 150 µL of urine supernatant was spiked with internal standard and diluted 1:1 with 2% formic acid. Oasis WAX SPE columns were conditioned with 1mL methanol, then equilibrated with 1mL of water, prior to addition of the 300µL sample (vacuum sufficient to achieve 1ml/min). The columns were then washed with 1mL 2% formic acid, and then eluted with 1ml of methanol then 300µL of 5% (w/v) ammonium hydroxide in methanol (x2). The resulting eluate was evaporated to dryness under nitrogen before reconstitution in 50µL mobile phase.

*UHPC-MS/MS analyses*

Experiments were performed on a Quattro Premier spectrometer (Waters Corporation, Manchester, UK), operated in negative ion mode with the ESI capillary voltage set to 3kV and the cone voltage to 60V. The nitrogen desolvation and cone gas flow rates were set to 1000 and 50Lh^−1^ respectively, with a source temperature of 120°C and desolvation gas temperature set to 300°C. The mass spectrometer was operated in multiple reaction monitoring mode. For *cis,syn* T<>T three transitions were identified: 253>210, 253>210 and 253>139 ([^2^H_8_]-T<>T: 261>159). For *cis,syn* T<>pT, three mass transitions were identified 545>447, 545>253, and 545>195 (([U-^13^C_10_, U-^15^N_2_]-T<>pT: 557>431, 557>333, 557>298, and 557>125). Conditions for each was as follows: entry and exit voltage of 30eV with a collision energy of 31eV and an Argon collision cell pressure of 5.9e^-003^. Masslynx version 4.1 (Waters Corporation, Manchester, UK) was used to control the IM-MS instrument and for data acquisition and processing. UHPLC was performed on a Waters Acquity system (Waters Corporation, Manchester, UK) with an Acquity UHPLC HSS T3® (Waters Corporation, Manchester, UK) column (2.1 x 50mm). The UHPLC system was coupled to a post-column T-piece fitting (Waters Corporation, Manchester, UK) which was also connected to a further Dionex HPLC system (Thermo Fisher UK Ltd, Hemel Hempstead, UK) with the outlet going to the ESI ion source of the mass spectrometer. Cleaned up urine samples (5µL injected) were eluted with the following gradient: 99.9% A (0-2min), increased to 30% B (2-4min) and then to 99.9% A (4-6min), where A=2.5mM ammonium formate and B=acetonitrile. The mobile phase flow rate was set to 0.1mL/min. The additional HPLC flow rate was set to 0.1mL/min employing an isocratic flow of acetonitrile to aid ionisation. The limit of detection was 396fmol.

**Supplementary References**

Liu F-T, Yang NC. Photochemistry of cytosine derivatives. 1. Photochemistry of thymidylyl-(3’5’)-deoxycytidine. *Biochemistry* 1978; **17**:4865-4876.

Podmore ID, Cooke MS, Herbert KE *et al.* Quantitative Determination of Cyclobutane Thymine Dimers in DNA by Stable Isotope-Dilution Mass Spectrometry. *Photochem Photobiol* 1996; **64**:310-315.

Wang SY. Photochemical reactions in frozen solutions. *Nature* 1961; **190**:690-694.
